# Onkologische Operationsberichte: Minimalanforderungen, rechtliche Aspekte und zukünftige Entwicklungen

**DOI:** 10.1007/s00104-025-02321-z

**Published:** 2025-06-16

**Authors:** Johannes Klose, Sandra Böhm, Christiane J. Bruns, Paul Chojecki, Lena-Christin Conradi, Stefan Fichtner-Feigl, Robert Grützmann, Jörg Heberer, Boris Jansen-Winkeln, Jens Jakob, Kay Kohlhaw, Astrid Oehme, Ulrich Ronellenfitsch, Susanne Roth, Christoph Rüger, Igor Sauer, Harald Schulz, Sven Zehnder, Jörg Kleeff

**Affiliations:** 1 Klinik für Viszerale, Gefäß- und Endokrine Chirurgie, Universitätsmedizin Halle (Saale), Martin-Luther-Universität Halle-Wittenberg, Ernst-Grube-Str. 40, 06120 Halle (Saale), Deutschland; 2 HFC Human Factors-Consult, Berlin, Deutschland; 3 Klinik und Poliklinik für Allgemein‑, Viszeral‑, Thorax- und Transplantationschirurgie, Universität Köln, Köln, Deutschland; 4 Fraunhofer Heinrich-Hertz-Institut, Berlin, Deutschland; 5 Klinik für Allgemein‑, Viszeral- und Kinderchirurgie, Universitätsmedizin Göttingen, Göttingen, Deutschland; 6 Klinik für Allgemein- und Viszeralchirurgie, Universitätsklinikum Freiburg, Freiburg, Deutschland; 7 Chirurgische Klinik, Universitätsklinikum Erlangen, Erlangen, Deutschland; 8 Rechtsanwaltskanzlei Heberer & Kollegen, München, Deutschland; 9 Klinik für Allgemein‑, Viszeral‑, Thorax- und Gefäßchirurgie, Klinikum St. Georg, Leipzig, Deutschland; 10 Chirurgische Klinik, Universitätsmedizin Mannheim, Mannheim, Deutschland; 11 Klinik für Allgemein‑, Viszeral‑, MIC- und Gefäßchirurgie, Sana Klinikum Borna, Borna, Deutschland; 12 Universitätsklinik für Allgemeine‑, Viszeral- und Transplantationschirurgie, Universitätsklinikum Tübingen, Tübingen, Deutschland; 13 Chirurgische Klinik, Charité Universitätsmedizin Berlin, Berlin, Deutschland

**Keywords:** OP-Bericht, Onkologie, Qualitätsstandard, Recht, KI, Operation report, Oncology, Quality standard, Law, Artificial intelligence

## Abstract

Die Etablierung onkologischer Zentren zur interdisziplinären Versorgung krebskranker Patienten beruht auf definierten Kriterien, die sich einer regelmäßigen externen Prüfung unterziehen müssen. Dadurch soll ein einheitlicher Qualitätsstandard in der onkologischen Versorgung in Deutschland gesichert sein. Die Qualität der chirurgischen Leistung wird dabei unter anderem an der Anzahl der behandelten Fälle oder der Erfahrung ausgewählter Operateure definiert. Die erstellten Operationsberichte sind jedoch sehr heterogen, da für die Zertifizierung zu einem onkologischen Zentrum keine Vorgaben erfüllt werden müssen. Die Assoziation Chirurgische Onkologie (ACO) hat sich daher zum Ziel gesetzt, einen Minimalstandard für einen einheitlichen onkologischen Operationsbericht vorzuschlagen. Neben den im Operationsbericht zu erwähnenden Aspekten – beispielhaft bezogen auf die einzelnen Organsysteme – sollen auch rechtliche Aspekte sowie der Einsatz künstlicher Intelligenz näher betrachtet werden.

## Abstract (en)

The establishment of comprehensive oncological centers for the interdisciplinary care of cancer patients is based on defined criteria that must undergo regular external review to ensure a uniform quality standard of cancer care in Germany. The quality of surgical performance is defined, among other factors, by the number of cases treated or the experience of individual surgeons; however, the operation reports made are highly heterogeneous, as no specific requirements need to be met for certification as an oncological center. The German Association for Surgical Oncology (ACO) has therefore set the goal of proposing a minimum standard for oncological operation reports. In addition to the aspects to be included in the operation reports, exemplified with respect to individual organ systems, legal considerations and the use of artificial intelligence should also be examined in greater detail.

Die Grundvoraussetzungen zur Sicherung der Ergebnisqualität nach onkologischer Chirurgie der Organe des Verdauungstraktes ist ein standardisiertes, interdisziplinär und individuell auf den jeweiligen Patienten abgestimmtes Vorgehen. Moderne onkologische Chirurgie sollte daher vorzugsweise an durch die Deutsche Krebsgesellschaft (DKG) oder die Deutsche Gesellschaft für Allgemein- und Viszeralchirurgie (DGAV) zertifizierten Zentren für das jeweilige Organsystem bzw. die jeweilige Tumorerkrankung durchgeführt werden. Bedingt durch das Einhalten der geforderten Mindeststandards, kann die Behandlung von onkologischen Patienten damit mit hoher Sicherheit und onkologischer Qualität durchgeführt werden [1, 2]. In diesem Zusammenhang konnte gezeigt werden, dass Patienten, die in zertifizierten Zentren behandelt wurden, einen Überlebensvorteil gegenüber Patienten hatten, die nicht in einem zertifizierten Zentrum behandelt wurden [3–6]. Für die optimale Ergebnisqualität sind alle beteiligten Disziplinen und Berufsgruppen wichtig, also neben der Chirurgie auch die Gastroenterologie, Onkologie, Strahlentherapie, interventionelle Radiologie, Pathologie, Psychoonkologie, Physiotherapie etc. [7].

Gegenwärtig existieren noch keine Mindestanforderungen oder Standards für onkologische OP-Berichte durch die jeweiligen Fachgesellschaften. Dabei stellt die fachgerechte Durchführung der onkologischen Operationen und deren Dokumentation einen wichtigen Bestandteil in der erfolgreichen Behandlung von an Krebs erkrankten Patienten dar. Basierend auf der Standardisierung des OP-Berichts, ist auch ein Mindeststandard für onkologische Operationen anzustreben. Zusätzlich kann ein standardisierter OP-Bericht als Vorlage und Qualitätssicherung die Ausbildung nachfolgender Operateure verbessern.

Das Ziel dieses Papiers der Assoziation Chirurgische Onkologie (ACO) und der organspezifischen Arbeitsgruppen der DGAV (CAGLP/CAOGI/CACP) ist es, Mindestanforderungen für onkologische OP-Berichte aus dem Bereich der Viszeralchirurgie zu definieren. Ziel ist *nicht*, alle operativen Details für die jeweiligen Tumorentitäten zu beschreiben, sondern die wichtigsten Informationen zu definieren, die ein onkologischer OP-Bericht enthalten sollte. Darüber hinaus werden die rechtlichen Grundlagen diskutiert. Abschließend wird beschrieben, wie in Zukunft mithilfe der künstlichen Intelligenz eine standardisierte Erstellung (onkologischer) OP-Berichte aus unserem Fachgebiet möglich werden könnte.

Einen wichtigen Bestandteil von OP-Berichten sollte auch die Beschreibung intraoperativer Komplikationen darstellen. Intraoperative Komplikationen sind mit postoperativen Komplikationen und konsekutiv erhöhter Morbidität und Mortalität assoziiert [8–10]. Beispielsweise kann die Einteilung der intraoperativen Komplikationen nach der „ClassIntra“-Klassifikation erfolgen [11], die auf einer prospektiv angelegten Untersuchung basiert und deren Validität inzwischen bestätigt werden konnte (s. Tab. 1; [12, 13]). Die Klassifikation beschreibt jedwede Abweichungen vom normalen oder „idealen“ Verlauf einer Operation. Dies beginnt etwa bei einer Serosaverletzung des Darms und schließt neben Blutungen aus kleinen und großen Gefäßen auch systemische Komplikationen wie das Auftreten einer Arrhythmie mit ein [13]. *Selbstverständlich muss jedoch nicht zwingend eine einheitliche Klassifikation an dieser Stelle verwendet werden.*

**Tab. 1:** Einteilung der intraoperativen Komplikationen. (Adaptiert nach [11])

Grad	Definition	Beispiele
0	Keine Abweichung vom idealen intraoperativen Verlauf	–
I	Jedwede Abweichung vom idealen intraoperativen Verlauf: – Ohne Notwendigkeit weiterer kleiner Maßnahmen oder Interventionen – Patienten klinisch ohne oder mit nur milden Symptomen	Blutung: überdurchschnittliche Blutung aus kleinkalibrigen Gefäßen, selbstlimitierend oder mit Koagulation versorgbar
		Verletzungen: minimale Serosadefekte, die keiner weiteren Behandlung bedürfen
		Kauterisierung: minimale Verbrennungen an der Haut
		Arrhythmie: Extrasystolen ohne kardiozirkulatorische Relevanz
II	Jedwede Abweichung vom idealen intraoperativen Verlauf: – Mit Notwendigkeit weiterer kleiner Maßnahmen oder Interventionen – Patienten klinisch mit moderater Symptomatik, die nicht lebensgefährlich oder mit dauerhaften Folgeschäden assoziiert ist	Blutung: aus mittelgroßen Arterien oder Venen, versorgbar mittels Ligatur; Applikation von Tranexamsäure
		Verletzung: nichttransmurale Darmwandverletzungen, die einer Übernaht bedürfen
		Kauterisierung: moderate Verbrennungen der Haut, die eine nichtinvasive Wundbehandlung benötigen
		Arrhythmie: Rhythmusstörung, die einer medikamentösen Therapie bedarf ohne kardiozirkulatorische Wirkung
III	Jedwede Abweichung vom idealen intraoperativen Verlauf: – Mit Notwendigkeit weiterer moderater Maßnahmen oder Interventionen – Patienten mit schwerer Symptomatik, die potenziell lebensbedrohlich oder mit dauerhaften Folgeschäden assoziiert ist	Blutung: aus großkalibrigen Arterien oder Venen mit transienter hämodynamischer Instabilität, versorgbar mit Ligatur oder Naht; Bluttransfusion
		Verletzung: transmurale Darmwandverletzungen, die einer Resektion bedürfen
		Kauterisierung: schwere Verbrennungen der Haut, die ein chirurgisches Débridement benötigen
		Arrhythmie: Rhythmusstörung, die einer medikamentösen Therapie bedarf mit transienter kardiozirkulatorischer Wirkung
IV	Jedwede Abweichung vom idealen intraoperativen Verlauf: – Mit Notwendigkeit weiterer großer dringender Maßnahmen oder Interventionen – Patienten mit lebensbedrohlicher oder mit dauerhaften Folgeschäden assoziierter Symptomatik	Blutung: lebensbedrohliche Blutungen mit Splenektomie; Massentransfusion; Intensivaufenthalt
		Verletzung: Verletzungen zentraler arterieller oder venöser Gefäße, die einer Darmresektion bedürfen
		Kauterisierung: lebensbedrohliche Verbrennungen mit Flammenbildung, die einer intensivmedizinischen Überwachung bedürfen
		Arrhythmie: Rhythmusstörungen, die elektrisch kardiovertiert oder defibrilliert oder intensivmedizinisch therapiert/überwacht werden müssen
V	Jedwede Abweichung vom idealen intraoperativen Verlauf mit intraoperativem Versterben des Patienten	–

## Juristische Anforderungen an Form und Inhalt eines OP-Berichtes

Der *Zweck* der Dokumentation ist die* Qualitätssicherung*, Informationen für Mit- und Weiterbehandler, die Rechenschaftslegung gegenüber Patienten und der Nachweis der Leistungserbringung zur korrekten Abrechnung.

### *Umfang und Inhalt* der Dokumentation

Hinsichtlich des Umfangs ist gemäß der BGH-Rechtsprechung basierend auf § 630f BGB („sämtliche aus fachlicher Sicht für die derzeitige und künftige Behandlung wesentlichen Maßnahmen und deren Ergebnisse […] aufzunehmen“) die ausführliche, sorgfältige und vollständige Dokumentation der Operation einschließlich der pflegerischen Maßnahmen vorzunehmen.

Laut Rechtsprechung müssen „Operationsberichte […] für den fachkundigen Dritten selbsterklärend sein […]. Sie müssen lesbar, in sich widerspruchsfrei und nachvollziehbar“ sein (LSG Baden-Württemberg, Urteil v. 25.09.2013, Az. L 5 KA 3347/11) [22].

Maßstab für den Inhalt der Dokumentation ist nach ständiger Rechtsprechung stets die nach dem Facharztstandard medizinische Üblichkeit und Erforderlichkeit. Ist die Dokumentation einer Maßnahme aus medizinischer Sicht nicht erforderlich, so ist sie aus Rechtsgründen auch nicht geboten. „Maßnahmen sind nur dann in den Krankenunterlagen zu dokumentieren, wenn dies erforderlich ist, um Ärzte und Pflegepersonal über den Verlauf der Krankheit und die bisherige Behandlung im Hinblick auf künftige medizinische Entscheidungen ausreichend zu informieren. Ein Operationsbericht muss eine stichwortartige Beschreibung der jeweiligen Eingriffe und Angaben über die hierbei angewandte Technik enthalten. Nicht erforderlich ist hingegen die Wiedergabe von medizinischen Selbstverständlichkeiten […]“ (OLG Oldenburg, Urteil v. 30.01.2008, Az. 5 U 92/06) [23]. Spezielle Rechtsvorschriften, z. B. Qualitätssicherungsrichtlinien, können zudem ausdrücklich bestimmte Angaben fordern.

Gemäß juristischen Empfehlungen (Heberer, BDC-Merkblatt „Juristische Anforderungen an einen OP-Bericht“) [24] ergibt sich, basierend auf dem Stand der Rechtsprechung, folgender notwendiger, jedoch nicht abschließender Inhalt:

Die wesentlichen, für eine spätere ärztliche Beurteilung voraussichtlich unerlässlichen Fakten über den Verlauf der OP, insbesondere regelmäßig

- etwaige spezielle Lagerung,
- Operationssitus,
- OP-Methode mit stichwortartiger Beschreibung (OLG Oldenburg, Az. 5 U 92/06), Gründe für das Abweichen von einer herkömmlichen OP-Methode,
- wesentliche Arbeitsschritte im Einzelnen,
- Befunde während des OP-Verlaufs; negative Befunde nur, wenn ein medizinischer Anlass dazu besteht,
- jede Abweichung vom Normalverlauf, Komplikationen, unerwartete Zwischenfälle, jede Besonderheit, Erweiterungen der OP,
- Wechsel des Operateurs oder im OP-Team sowie der Status des Patienten beim Wechsel,
- bei „Anfängeroperationen“ möglichst umfassende Dokumentation; bei selbstständiger Operation eines sich noch in der Facharztausbildung befindlichen Arztes ist auch routinemäßiges Vorgehen zu dokumentieren,
- Anweisungen zu postoperativem Procedere.

### *Zeitpunkt* der Dokumentation

Gemäß § 630f BGB ist die Behandlungsdokumentation „in unmittelbarem zeitlichem Zusammenhang mit der Behandlung“ zu erstellen. Laut Rechtsprechung ist hierfür ein „Zeitraum, in dem dem Arzt die Einzelheiten der Behandlung noch präsent sind“ (KG, Urteil vom 10.01.2013, Az.: 20 U 225/10, OVG Münster, Urteil v. 25.11.2015, Az: 6t A 2679/13) maßgeblich, eine feste zeitliche Grenze existiert nicht [25, 26]. Entscheidend sind nämlich stets die konkreten Umstände des Einzelfalles. Einigkeit besteht jedoch in der Rechtsprechung dahingehend, dass je mehr Zeit zwischen OP und Erstellung des OP-Berichtes verstreicht, umso mehr steigt die Gefahr des Verlustes des Beweiswerts des OP-Berichts.

### *Form* der Dokumentation

**Abb. 1:**
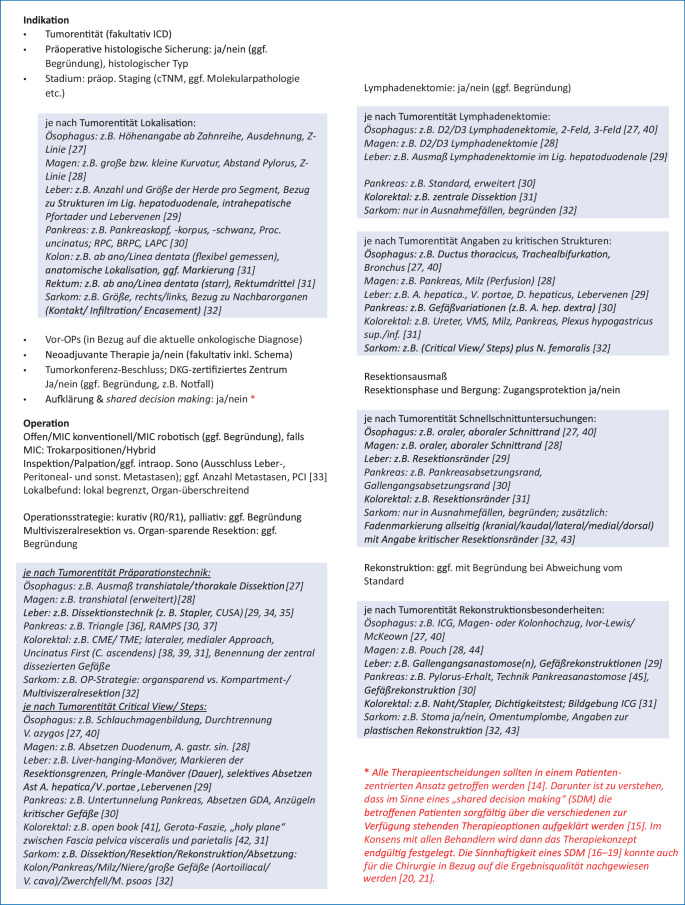
Mindeststandard onkologischer OP-Bericht

Die Form eines OP-Berichtes ist nicht gesetzlich festgelegt. Aus § 630f BGB ergibt sich, dass Patientenakten und somit auch OP-Berichte als deren Bestandteil „in Papierform oder elektronisch“ geführt werden können. Zulässig, teilweise auch notwendig nach Qualitätssicherungsvorschriften, sind auch zusätzliche Bild- oder Videoaufzeichnungen. Bei Berichtigungen und Änderungen müssen sowohl die ursprüngliche Dokumentation als auch der Zeitpunkt der Änderung erkennbar sein (Abb. 1).

### *Intraoperative „adverse events“* (iAEs)

*Fotodokumentation*, fakultativ, sofern eine Annotation durch die Chirurgie erfolgt und eine digitale Speicherung möglich ist:

- Resektat ggf. mit Markierung kritischer Resektionsränder,
- Situs nach Resektion vor Rekonstruktion,
- Situs nach Rekonstruktion,
- Besonderheiten intraoperativ.

### Bilddokumentation

Die intraoperative Bilddokumentation nimmt durch die zunehmende routinemäßige Anwendung minimal-invasiver Operationstechniken einen immer größeren Stellenwert ein.

Auch wenn hier wenig zitierfähige Literatur vorhanden ist, gehört in vielen Kliniken die intraoperative Fotodokumentation des Befundes oder kritischer Strukturen im Rahmen eines minimal-invasiven Eingriffs zum Standard [46, 47]. Ferner ist es üblich, dass robotische Operationen komplett oder teilweise als Video aufgezeichnet werden, sodass auch hier eine digitale Bilddokumentation vorhanden ist. Darüber hinaus werden in zahlreichen Kliniken auch die Resektate fotografiert und die Aufnahmen archiviert.

*Eine Bild- oder Videodokumentation der Operationen kann aus juristischer Sicht an Bedeutung gewinnen*, um zum einen anhand der ordnungsgemäßen Dokumentation im Rahmen von Klagen wegen etwaiger Behandlungsfehler eine Lege-artis-Behandlung beweisen zu können und zum anderen der Gefahr einer Beweislastumkehr zu begegnen, die droht, wenn eine medizinisch gebotene wesentliche Maßnahme und ihr Ergebnis nicht dokumentiert werden. Ferner muss eine solche Bild- oder Videodokumentation erfolgen, wenn diese in anderen Rechtsgrundlagen, wie z. B. Qualitätssicherungsrichtlinien, gefordert wird.

Für zahlreiche Studien ist darüber hinaus die Möglichkeit der Dokumentation der minimal-invasiv durchgeführten Operation für den Studieneinschluss der Patienten bzw. die Zulassung als Studienzentrum verpflichtend, da anhand dieser Dokumentation die chirurgische Qualität beurteilt werden kann. Es ist daher keineswegs nur spekulativer Natur, dass diese Form der Bild- und Videodokumentation auch einen Einzug in die tägliche klinische Routine in Deutschland erfahren wird.

## Ausblick: künstliche Intelligenz zur Generierung standardisierter OP-Berichte

Aktuelle Forschungsarbeiten betonen den hohen Dokumentationsaufwand im Krankenhaus und untersuchen KI-basierte Lösungen zur Reduzierung dieses Aufwandes, der zu Überlastung und schlechterer Versorgungsqualität beitragen kann [48, 49]. Vielversprechende Ansätze wie der DAX Copilot zeigen, dass durch den Einsatz von KI der Zeitaufwand für Dokumentation verringert und die Qualität der persönlichen Begegnungen zwischen medizinischem Personal und Patienten verbessert werden kann [50]. Zu den identifizierten Potenzialen zur Aufwandsreduktion gehören medizinische Schreibkräfte, Workflow-Optimierungen und technologische Lösungen [51].

Aktuell verfügbare Systeme wie Nuance (Burlington, Massachusetts, USA) und DeepScribe (San Francisco, Kalifornien, USA) nutzen Spracherkennungstechnologien, um gesprochene Sprache in präzise Dokumentation umzuwandeln. Systeme zur Videodokumentation wie Caresyntax und Proximie integrieren den Operationssaal mit Krankenhausinformationssystemen (KIS) und bieten erweiterte Analysen, um die Produktivität und Ergebnisse von Operationen zu verbessern. Trotz dieser Fortschritte bleibt die automatische Erfassung von OP-Situationen eine Herausforderung, die derzeit nur unzureichend adressiert wird. Dabei könnte eine (Teil‑)Automatisierung der OP-Bericht-Erstellung durch die Integration von KI-Bildverarbeitung und Spracheingabe mit innovativen Benutzerschnittstellen medizinische und administrative Prozesse optimieren.

Beispielhaft untersucht im Projekt KIARA (BMBF-Förderung, FKZ 16SV9036) ein interdisziplinärer Verbund die Umsetzung und Auswirkungen einer KI-basierten Erfassung von Arbeitsprozessen im OP zur automatisierten Erstellung von OP-Berichten. Im Fokus stehen 2 exemplarische Prozeduren: die Nierentransplantation und die Mandibularrekonstruktion. Technisch konzentriert sich das Projekt auf die visuelle Verarbeitung mittels KI, um Instrumente und Arbeitsschritte zu detektieren und daraus dokumentationswürdige Ereignisse abzuleiten. Die Analyse dieser komplexen OP-Situationen erfolgt auf 2D-Kamerabildern, die von Standard-OP-Hardware wie OP-Leuchten-Kameras erfasst werden. Die Bildverarbeitung erfolgt durch Objekterkennungstechniken wie YOLO und visuell-linguistische Modelle wie CLIP oder BLIP. Neurosymbolische Ansätze, Graph-basierte Frameworks und „Human in the Loop“-Verfahren werden integriert, um die Qualität der Situationsanalysen zu optimieren.

Eine grafische intraoperative Nutzerschnittstelle wird entwickelt, um die Detektionen nachvollziehbar darzustellen und ggf. Korrekturschleifen abzubilden. Intraoperative Sprachbefehle können ergänzend genutzt werden, um visuell schwer detektierbare Informationen festzuhalten. Die postoperative Schnittstelle erleichtert die Überprüfung und Nachbearbeitung des Berichts durch die Operierenden, die weiterhin für dessen Richtigkeit verantwortlich bleiben. Die Einbettung des Systems in bestehende Arbeitsabläufe ist entscheidend, um die Akzeptanz zu erhöhen und die Effizienz der OP-Dokumentation zu steigern.

Der Einsatz von KI-Technologie soll das medizinische Personal nach der OP entlasten, indem ein automatisch generierter Berichtsentwurf bereitgestellt wird. Es muss aber berücksichtigt werden, dass der Einsatz von KI während einer OP die Arbeit aller OP-Teammitglieder beeinflusst. Deshalb ist eine partizipative Einbindung aller relevanten Nutzergruppen einschließlich Chirurgie, Pflege, Anästhesie, Patienten und Krankenhaus-IT entscheidend, um Akzeptanz, Zufriedenheit und Vertrauen in die KI-Systeme zu gewährleisten. Die Interaktion mit der KI erfordert eine benutzerfreundliche und ablenkungsarme Gestaltung der oben genannten Schnittstellen. Ethisch-rechtliche Fragen zur Datenerhebung und -nutzung sowie die Genauigkeitsparameter der KI-Dokumentation müssen geklärt werden einschließlich der Speicherung, des Zugriffs und der Auswirkungen auf die Arbeitsleistung [50, 52]. Die Verwendung offener Systeme bzw. Entwicklung eigener „Hilfskonstrukte“ mit Verwendung von Servern außerhalb Deutschlands bzw. der EU muss sehr kritisch gesehen werden. Transparenz in diesen Prozessen ist notwendig, um das Vertrauen der Nutzer in die Technologie zu sichern und ihre Akzeptanz zu fördern.

Die Nutzung von KI in der OP-Berichtsgenerierung verspricht eine erhebliche Effizienzsteigerung, indem sie den zeitlichen Aufwand reduziert und die medizinische Dokumentation zeitnah sowie präziser und konsistenter gestaltet. Durch die Integration in Krankenhausinformationssysteme können KI-Systeme nahtlos in bestehende Arbeitsabläufe eingebunden werden, was den gesamten Behandlungsprozess optimiert. Zukünftig könnte KI personalisierte Berichte erstellen, die auf die spezifischen Bedürfnisse unterschiedlicher Nutzergruppen zugeschnitten sind. Gleichzeitig müssen ethische und rechtliche Aspekte berücksichtigt werden, insbesondere in Bezug auf Datenschutz und Datensicherheit. Langfristig ist die Akzeptanz durch medizinische Fachkräfte entscheidend, die durch kontinuierliche Schulungen und Einbindung in die Systementwicklung gefördert wird.

## Fazit

In diesem Artikel werden Mindestanforderungen für das Erstellen eines standardisierten und einheitlichen onkologischen Operationsberichts definiert. Essenziell dafür ist zunächst die Beachtung der juristischen Rahmenbedingungen. Als Blaupause für das Erstellen eines individuellen onkologischen Operationsberichts sollen die rechtfertigende Indikation sowie die Operationsschritte dienen. Hier sollten die Operationstechnik, das Ausmaß der Resektion und Lymphadenektomie, die Radikalität des Eingriffs und die anschließende Rekonstruktion präzise erläutert werden. Es sollte sich an den Definitionen der nationalen oder internationalen Leitlinien bzw. konsensuellen Empfehlungen orientiert werden. Zudem ist ein Verweis auf das Auftreten intraoperativer Komplikationen notwendig, auch um potenziellen Rechtsstreitigkeiten begegnen zu können. Hier wird insbesondere auf die Bedeutung der Foto- und Videodokumentation hingewiesen. In diesem Artikel wird abschließend ein Ausblick auf das Potenzial künstlicher Intelligenz beim Erstellen eines onkologischen Operationsberichts skizziert. Neben einer Effizienzsteigerung ist auch eine Qualitätsverbesserung *möglich*, die allgemeinhin das oberste Ziel in der Versorgung krebskranker Patienten sein sollte.
